# Distinct Patterns of Constitutive Phosphodiesterase Activity in Mouse Sinoatrial Node and Atrial Myocardium

**DOI:** 10.1371/journal.pone.0047652

**Published:** 2012-10-15

**Authors:** Rui Hua, Andrew Adamczyk, Courtney Robbins, Gibanananda Ray, Robert A. Rose

**Affiliations:** Department of Physiology and Biophysics, Faculty of Medicine, Dalhousie University, Halifax, Nova Scotia, Canada; Brigham & Women's Hospital – Harvard Medical School, United States of America

## Abstract

Phosphodiesterases (PDEs) are critical regulators of cyclic nucleotides in the heart. In ventricular myocytes, the L-type Ca^2+^ current (I_Ca,L_) is a major target of regulation by PDEs, particularly members of the PDE2, PDE3 and PDE4 families. Conversely, much less is known about the roles of PDE2, PDE3 and PDE4 in the regulation of action potential (AP) properties and I_Ca,L_ in the sinoatrial node (SAN) and the atrial myocardium, especially in mice. Thus, the purpose of our study was to measure the effects of global PDE inhibition with Isobutyl-1-methylxanthine (IBMX) and selective inhibitors of PDE2, PDE3 and PDE4 on AP properties in isolated mouse SAN and right atrial myocytes. We also measured the effects of these inhibitors on I_Ca,L_ in SAN and atrial myocytes in comparison to ventricular myocytes. Our data demonstrate that IBMX markedly increases spontaneous AP frequency in SAN myocytes and AP duration in atrial myocytes. Spontaneous AP firing in SAN myocytes was also increased by the PDE2 inhibitor erythro-9-[2-hydroxy-3-nonyl] adenine (EHNA), the PDE3 inhibitor milrinone (Mil) and the PDE4 inhibitor rolipram (Rol). In contrast, atrial AP duration was increased by EHNA and Rol, but not by Mil. IBMX also potently, and similarly, increased I_Ca,L_ in SAN, atrial and ventricular myocytes; however, important differences emerged in terms of which inhibitors could modulate I_Ca,L_ in each myocyte type. Consistent with our AP measurements, EHNA, Mil and Rol each increased I_Ca,L_ in SAN myocytes. Also, EHNA and Rol, but not Mil, increased atrial I_Ca,L_. In complete contrast, no selective PDE inhibitors increased I_Ca,L_ in ventricular myocytes when given alone. Thus, our data show that the effects of selective PDE2, PDE3 and PDE4 inhibitors are distinct in the different regions of the myocardium indicating important differences in how each PDE family constitutively regulates ion channel function in the SAN, atrial and ventricular myocardium.

## Introduction

Phosphodiesterases (PDEs) are phosphohydrolase enzymes that are responsible for the degradation of the cyclic nucleotides adenosine and guanosine 3′,5′ cyclic monophosphate (cAMP and cGMP) [1], [2]. PDEs play critical roles in the modulation of cellular functions that depend on cAMP and cGMP in the heart, including electrical conduction, contractility, metabolism and transcription [3], by controlling the levels of these powerful signaling molecules in cells. PDEs exist in 11 families (PDE1-11) with several isoforms in each and are regulated by diverse mechanisms including phosphorylation, binding of cyclic nucleotides, calcium binding and protein-protein interactions [1]. With such a large number of families and isoforms PDE signaling is clearly complex and in this context it is now thought that PDEs are importantly involved in the compartmentation of cyclic nucleotide signaling whereby the subcellular localization of different PDE isoforms can lead to distinct spatial and temporal pools of cAMP and/or cGMP [3]–[6]. This can result in distinct roles for specific PDEs in different parts of the cell or in different conditions.

Among the PDE families expressed in the heart [7] PDE2, PDE3 and PDE4 have been shown to contribute substantially to cyclic nucleotide regulation, especially in the context of modulating ion channel function [3], [4], [8], [9]. Numerous studies have demonstrated that the L-type Ca^2+^ current (I_Ca,L_) is a critical target of regulation by PDEs in ventricular myocytes. Specifically, it is established that global PDE inhibition with the broad spectrum inhibitor 3-Isobutyl-1-methylxanthine (IBMX) potently increases basal ventricular I_Ca,L_ in mice and rats [8], [10], [11]. Interestingly, selective inhibition of PDE2, PDE3 or PDE4 alone has no effect on basal I_Ca,L_; however combined inhibition of PDE3 and PDE4 does increase basal I_Ca,L_ and inhibition of PDE2, PDE3 and PDE4 increases basal I_Ca,L_ very similarly to IBMX [8], [10], [11]. Although the effects of PDE2, 3 and 4 inhibition on I_Ca,L_ have been well characterized in ventricular myocytes much less is known about the role of these PDE families in the sinoatrial node (SAN) and atria, particularly in mice, a very common model organism due to its use in studies incorporating genetic manipulations.

**Figure 1:**
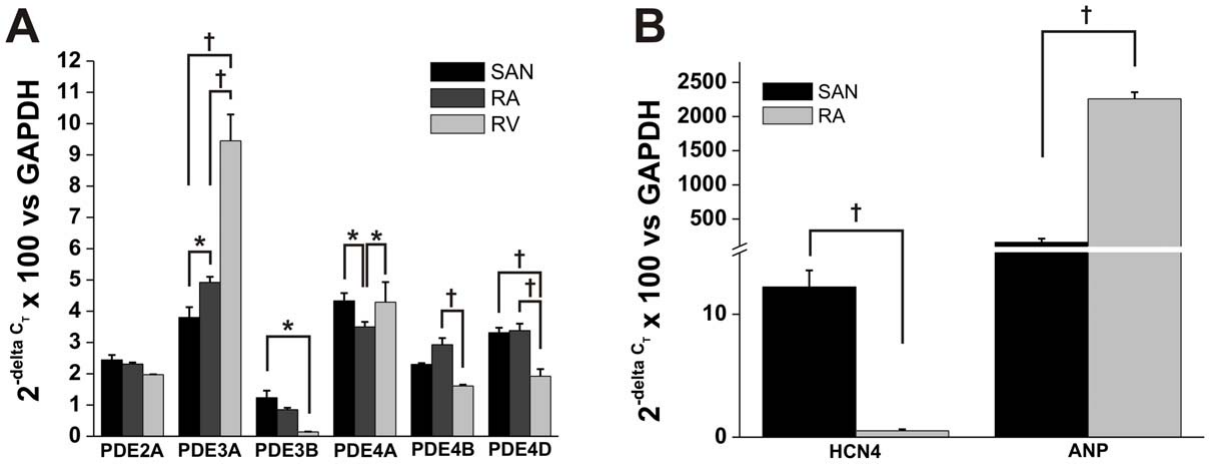
Quantitative mRNA expression of PDE 2, 3 and 4 subtypes in mouse SAN, right atrium and right ventricle. **A.** Expression of PDE2A, PDE3A, PDE3B, PDE4A, PDE4B and PDE4D are shown relative to GAPDH for the SAN, right atrium (RA) and right ventricular free wall (RV). **B.** SAN samples were distinguished from RA samples by the characteristic pattern of expression of HCN4 and ANP in these regions. SAN tissue shows high expression of HCN4 and low expression of ANP whereas the RA shows very low HCN4 expression and very high ANP expression. Note scale break on Y-axis. Data are means ± SEM; *n* = 5 SAN trials, 5 right atrial appendage trials and 3 right ventricular free wall trials; *P<0.05; ^†^ *P*<0.001 by two way ANOVA with Tukey's posthoc test.

The SAN contains the specialized pacemaker myocytes whose spontaneous activity is responsible for determining heart rate [12]. Spontaneous action potentials (APs) in these SAN myocytes are characterized by the presence of a diastolic depolarization (DD), during which the SAN myocyte gradually depolarizes until the threshold for an AP is reached [12]–[14]. Several ionic currents and mechanisms contribute to the generation of the DD including I_Ca,L_ [15]. Consistent with this hypothesis, it has recently been demonstrated that two forms of L-type Ca^2+^ channel, Ca_V_1.2 and Ca_V_1.3, contribute to total I_Ca,L_ in SAN myocytes, in contrast to ventricular myocytes which only express Ca_V_1.2 [16], [17]. Ca_V_1.3 channels activate at more negative membrane potentials than Ca_V_1.2 enabling them to contribute prominently to the generation of the DD and thereby to the frequency of spontaneous AP firing and heart rate control. I_Ca,L_ is also a critical determinant of AP morphology in working atrial myocytes [18]. Like SAN myocytes, Ca_V_1.2 and Ca_V_1.3 both contribute to total I_Ca,L_ in atrial myocytes [19]. Given the unique physiology of the SAN and atria it is possible that PDE regulation of AP properties and ion channels, including I_Ca,L_, is different in these tissues in comparison to the ventricles.

**Figure 2:**
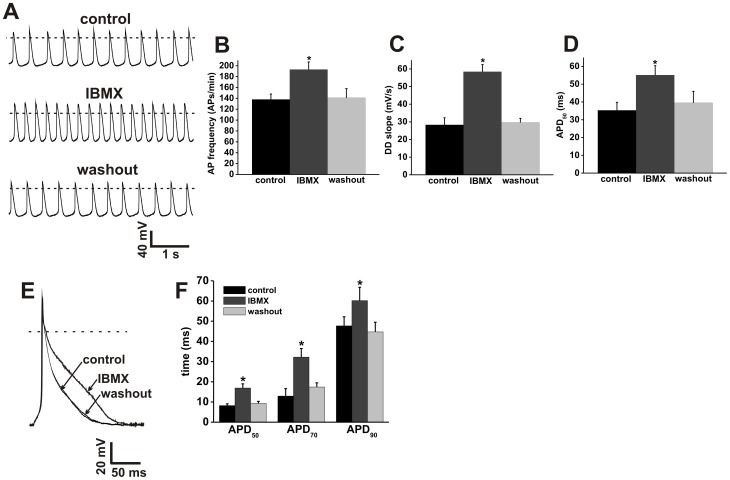
Effects of IBMX on action potential firing in SAN and atrial myocytes. **A.** Representative spontaneous AP recordings (5 s duration) in control conditions, in the presence of IBMX (100 µM) and after IBMX washout. Dotted lines are at 0 mV. Summary bar graphs illustrate the effects of IBMX on spontaneous AP frequency (**B**), DD slope (**C**) and APD_50_ (**D**). **E.** Representative stimulated right atrial myocyte APs in control conditions, in the presence of IBMX (100 µM) and after IBMX washout. Dotted line is at 0 mV. **F.** Summary of the effects of IBMX on atrial AP duration (APD_50_, APD_70_, and APD_90_). Summary data are means ± SEM; *n* = 11 SAN myocytes and 13 right atrial myocytes; *P<0.05 vs. control by one way ANOVA with Tukey's posthoc test.

The purpose of this study was to determine the roles of PDE2, PDE3 and PDE4 in regulating AP properties in mouse SAN and atrial myocytes. We also measured the effects of broad spectrum and family specific PDE inhibitors on basal I_Ca,L_ in SAN and atrial myocytes in comparison to ventricular myocytes. This work represents the first direct comparison of these inhibitors on I_Ca,L_ in these three regions of the myocardium in mice. Our data demonstrate that the patterns of constitutive PDE activity are distinct between SAN, atrial and ventricular myocytes, further adding to the complexity of PDE signaling in the heart.

**Figure 3:**
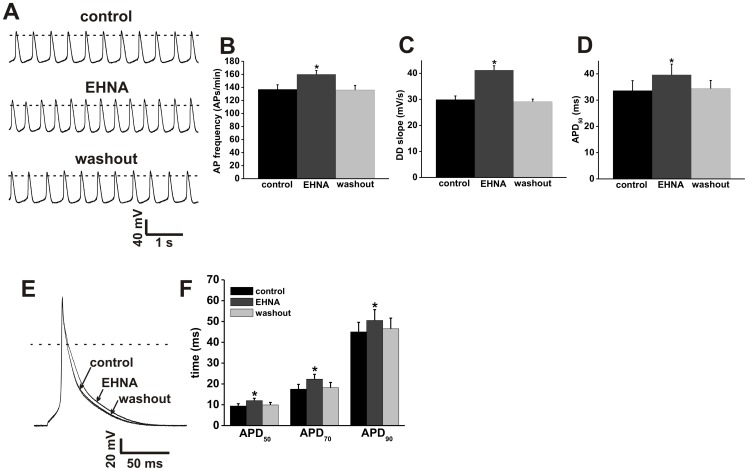
Effects of the PDE2 inhibitor EHNA on action potential firing in SAN and atrial myocytes. **A.** Representative spontaneous AP recordings (5 s duration) in control conditions, in the presence of EHNA (10 µM) and after EHNA washout. Dotted lines are at 0 mV. Summary bar graphs illustrate the effects of EHNA on spontaneous AP frequency (**B**), DD slope (**C**) and APD_50_ (**D**). **E.** Representative stimulated right atrial myocyte APs in control conditions, in the presence of EHNA (10 µM) and after EHNA washout. Dotted line is at 0 mV. **F.** Summary of the effects of EHNA on atrial AP duration (APD_50_, APD_70_, and APD_90_). Summary data are means ± SEM; *n* = 6 SAN myocytes and 7 right atrial myocytes; *P<0.05 vs. control by one way ANOVA with Tukey's posthoc test.

## Materials and Methods

### Ethics Statement

This study utilized male wildtype C57Bl/6 mice (Charles River) between the ages of 10–14 weeks. Experimental procedures were in accordance with the regulations of The Canadian Council on Animal Care and were approved by The Dalhousie University Committee on Laboratory Animals. In all experiments, mice were anesthetized by isoflurane inhalation and cervically dislocated before hearts were removed.

**Figure 4:**
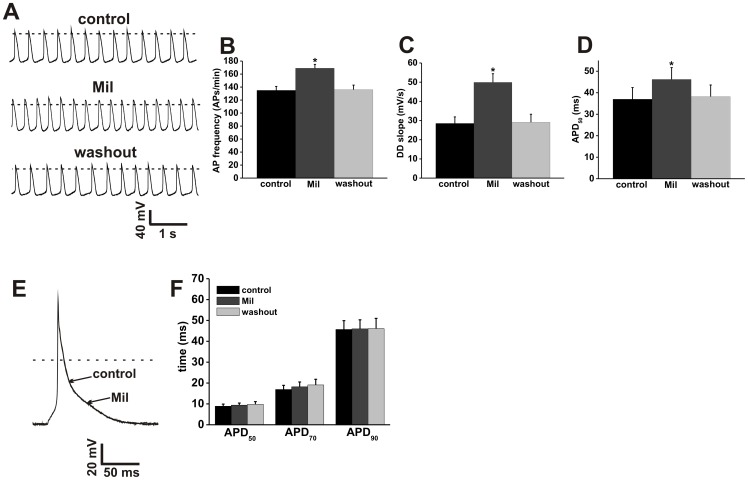
Effects of the PDE3 inhibitor milrinone on action potential firing in SAN and atrial myocytes. **A.** Representative spontaneous AP recordings (5 s duration) in control conditions, in the presence of Mil (10 µM) and after Mil washout. Dotted lines are at 0 mV. Summary bar graphs illustrate the effects of Mil on spontaneous AP frequency (**B**), DD slope (**C**) and APD_50_ (**D**). **E.** Representative stimulated right atrial myocyte APs in control conditions, in the presence of Mil (10 µM) and after Mil washout. Dotted line is at 0 mV. **F.** Summary of the effects of Mil on atrial AP duration (APD_50_, APD_70_, and APD_90_). Summary data are means ± SEM; *n* = 9 SAN myocytes and 9 right atrial myocytes; *P<0.05 vs. control by one way ANOVA with Tukey's posthoc test.

An expanded Materials and Methods section is available in Appendix S1.

### Experimental Approaches

Quantitative mRNA expression for PDE2A, PDE3A, PDE3B, PDE4A, PDE4B and PDE4D in mouse SAN, right atrium and right ventricular free wall was performed using methods we have described previously (see also Appendix S1) [20]. These specific PDE isoforms were selected because our electrophysiological experiments are focused on the PDE2, 3 and 4 families and prior studies [7], [11] have shown that the isoforms we measured are expressed in whole ventricular tissue and ventricular myocytes.

**Figure 5:**
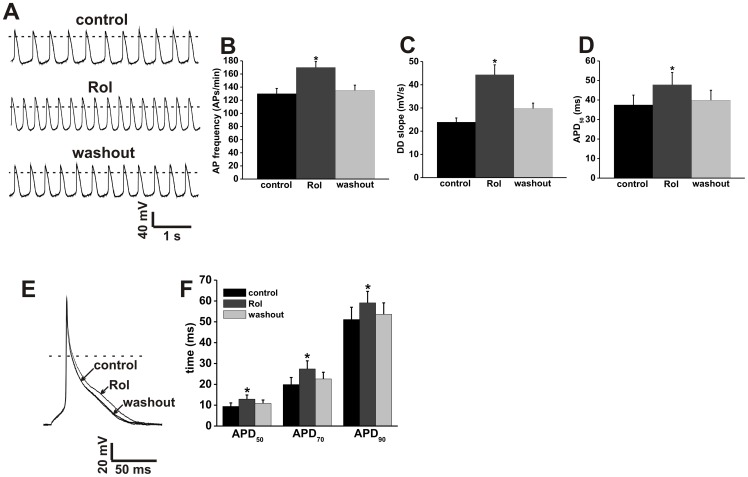
Effects of the PDE4 inhibitor rolipram on action potential firing in SAN and atrial myocytes. **A.** Representative spontaneous AP recordings (5 s duration) in control conditions, in the presence of Rol (10 µM) and after Rol washout. Dotted lines are at 0 mV. Summary bar graphs illustrate the effects of Rol on spontaneous AP frequency (**B**), DD slope (**C**) and APD_50_ (**D**). **E.** Representative stimulated right atrial myocyte APs in control conditions, in the presence of Rol (10 µM) and after Rol washout. Dotted line is at 0 mV. **F.** Summary of the effects of Rol on atrial AP duration (APD_50_, APD_70_, and APD_90_). Summary data are means ± SEM; *n* = 10 SAN myocytes and 7 right atrial myocytes; *P<0.05 vs. control by one way ANOVA with Tukey's posthoc test.

Mouse SAN, right atrial and right ventricular myocytes were isolated for patch-clamp recordings using procedures we have described previously [20]–[23]. Spontaneous and stimulated action potentials (APs) were recorded using the perforated patch-clamp technique in current clamp mode. I_Ca,L_ was recorded in the whole cell patch-clamp configuration. All recordings were performed at room temperature (22–23°C). The solutions and electrophysiological protocols for these experiments are provided in Appendix S1.

**Figure 6:**
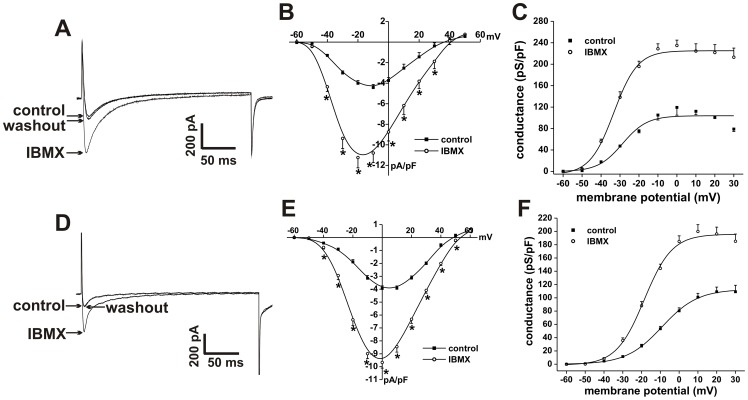
Effects of IBMX on L-type Ca^2+^ current in SAN and right atrial myocytes. **A.** Representative I_Ca,L_ recordings in SAN myocytes in control conditions, in the presence of IBMX (100 µM) and following IBMX washout. **B.** Summary I–V relationships for the effects of IBMX on SAN myocyte I_Ca,L_. **C.** Summary I_Ca,L_ conductance density plots for the effects of IBMX in SAN myocytes. **D.** Representative I_Ca,L_ recordings in right atrial myocytes in control conditions, in the presence of IBMX (100 µM), and following IBMX washout. **E.** Summary I–V relationships for the effects of IBMX on right atrial myocyte I_Ca,L_. **F.** Summary I_Ca,L_ conductance density plots for the effects of IBMX in right atrial myocytes. Summary data are means ± SEM; *n* = 8 SAN myocytes and 12 right atrial myocytes; *P<0.05 vs. control by paired Student's *t*-test.

### Statistics

All summary data are presented as means ± SEM. Data were analyzed by one or two way ANOVA with Tukey's posthoc analysis or paired Student's *t*-test. *P*<0.05 was considered significant.

**Figure 7:**
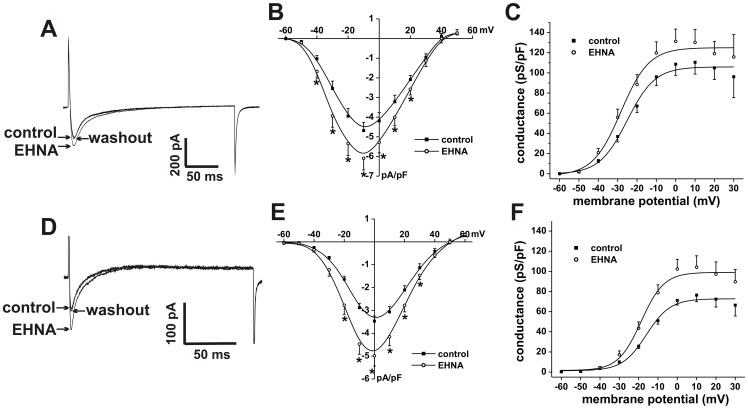
Effects of the PDE2 inhibitor EHNA on L-type Ca^2+^ current in SAN and right atrial myocytes. **A.** Representative I_Ca,L_ recordings in SAN myocytes in control conditions, in the presence of EHNA (10 µM) and following EHNA washout. **B.** Summary I–V relationships for the effects of EHNA on SAN myocyte I_Ca,L_. **C.** Summary I_Ca,L_ conductance density plots for the effects of EHNA in SAN myocytes. **D.** Representative I_Ca,L_ recordings in right atrial myocytes in control conditions, in the presence of EHNA (10 µM), and following EHNA washout. **E.** Summary I–V relationships for the effects of EHNA on right atrial myocyte I_Ca,L_. **F.** Summary I_Ca,L_ conductance density plots for the effects of EHNA in right atrial myocytes. Summary data are means ± SEM; *n* = 6 SAN myocytes and 10 right atrial myocytes; *P<0.05 vs. control by paired Student's *t*-test.

## Results

### Region specific mRNA expression of PDE2, 3 and 4 subtypes in the heart

Amongst the PDE2, 3 and 4 families, prior studies have shown that the PDE2A, PDE3A, PDE3B, PDE4A, PDE4B and PDE4D subtypes are expressed in whole ventricular myocardium and/or isolated ventricular myocytes in rodents [7], [11]. The relative expression of these PDE isoforms in the SAN and right atrial myocardium; however, have not been determined. Accordingly, we have measured the mRNA expression of PDE2A, PDE3A, PDE3B, PDE4A, PDE4B and PDE4D in the SAN, right atrium and right ventricular free wall using quantitative PCR (Figure 1A). Glyceraldehyde 3-phosphate dehydrogenase (GAPDH) was used as a reference gene. These three regions of myocardium coincide with the cell types used in our electrophysiology experiments (see below). SAN samples were distinguished from right atrial samples based on the high expression of HCN4 and low expression of ANP in the SAN compared to the atrium (Figure 1B) as previously described by us [20] and others [24].

**Figure 8:**
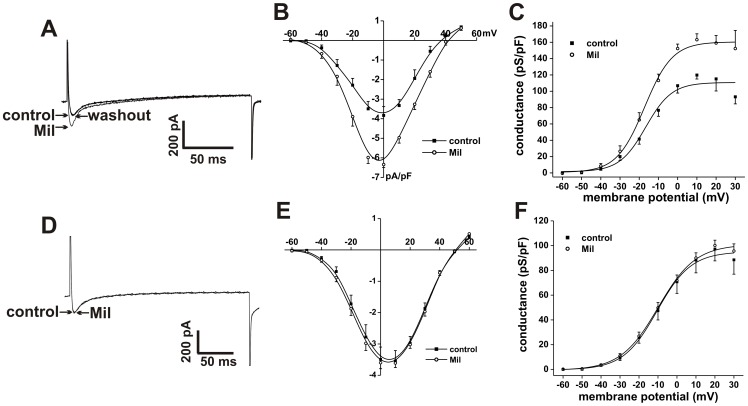
Effects of the PDE3 inhibitor milrinone on L-type Ca^2+^ current in SAN and right atrial myocytes. **A.** Representative I_Ca,L_ recordings in SAN myocytes in control conditions, in the presence of Mil (10 µM) and following Mil washout. **B.** Summary I–V relationships for the effects of Mil on SAN myocyte I_Ca,L_. **C.** Summary I_Ca,L_ conductance density plots for the effects of Mil in SAN myocytes. **D.** Representative I_Ca,L_ recordings in right atrial myocytes in control conditions, in the presence of Mil (10 µM), and following Mil washout. **E.** Summary I–V relationships for the effects of Mil on right atrial myocyte I_Ca,L_. **F.** Summary I_Ca,L_ conductance density plots for the effects of Mil in right atrial myocytes. Summary data are means ± SEM; *n* = 6 SAN myocytes and 12 right atrial myocytes; *P<0.05 vs. control by paired Student's *t*-test.

These data demonstrate that each region of the myocardium has a specific pattern of expression for the PDE2, 3 and 4 isoforms we have measured. Specifically, no differences were observed in the expression of PDE2A in each region of the myocardium; however, PDE3A, PDE3B, PDE4A, PDE4B and PDE4D all displayed significant differences in expression between SAN, right atrium and right ventricle as indicated in Figure 1A.

**Figure 9:**
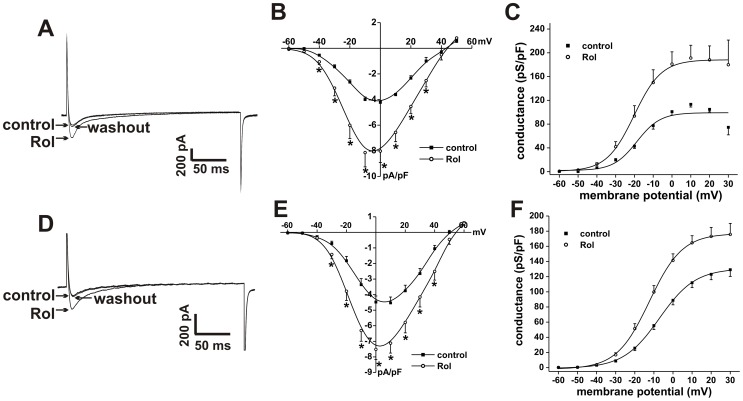
Effects of the PDE4 inhibitor rolipram on L-type Ca^2+^ current in SAN and right atrial myocytes. **A.** Representative I_Ca,L_ recordings in SAN myocytes in control conditions, in the presence of Rol (10 µM) and following Rol washout. **B.** Summary I–V relationships for the effects of Rol on SAN myocyte I_Ca,L_. **C.** Summary I_Ca,L_ conductance density plots for the effects of Rol in SAN myocytes. **D.** Representative I_Ca,L_ recordings in right atrial myocytes in control conditions, in the presence of Rol (10 µM), and following Rol washout. **E.** Summary I–V relationships for the effects of Rol on right atrial myocyte I_Ca,L_. **F.** Summary I_Ca,L_ conductance density plots for the effects of Rol in right atrial myocytes. Summary data are means ± SEM; *n* = 6 SAN myocytes and 12 right atrial myocytes; *P<0.05 vs. control by paired Student's *t*-test.

### Effects of global PDE inhibition on SAN and right atrial myocyte APs

IBMX is a broad spectrum PDE inhibitor that antagonizes all PDE isoforms [1], [11]. In our initial electrophysiological studies we measured the effects of IBMX (100 µM) on spontaneous AP firing in SAN myocytes and stimulated APs in right atrial myocytes (Figure 2; Tables S1 and S2). IBMX increased (*P*<0.05) spontaneous AP firing in SAN myocytes from 138±10 to 193±14 APs/min with no change in maximum diastolic potential (MDP; Figure 2B, Table S1). This change in AP frequency was associated with increases (*P*<0.05) in DD slope (28.3±4 mV/s in control vs. 58.4 4.1 mV/s in IBMX; Figure 2C) and APD_50_ (35.3±4.5 ms in control vs. 55.1±5.3 ms in IBMX; Figure 2D). Additional SAN AP parameters are shown Table S1, which also shows that the effects of IBMX on SAN myocyte AP firing are fully reversible upon washout.

In right atrial myocytes, IBMX increased (*P*<0.05) AP duration at 50, 70 and 90% repolarization (APD_50_, APD_70_, APD_90_) with no change in resting membrane potential (RMP; Figure 2F, Table S2). Specifically, APD_50_ was increased from 8.2±0.9 to 16.9±2.1 ms, APD_70_ was increased from 12.9±3.7 to 32.2±4.3 ms and APD_90_ was increased from 47.7±4.5 to 60.2±6.6 ms. Additional atrial AP parameters are shown in Table S2. The effects of IBMX on right atrial APs were reversed upon drug washout.

### Effects of selective PDE2, PDE3 and PDE4 inhibitors on SAN and right atrial myocyte APs

PDE2, PDE3 and PDE4 have been shown to be major contributors to total PDE activity in rodent ventricular myocytes [10], [11]; however, their relative contributions in mouse SAN and atrial myocytes are not known. Thus, we next studied the effects of the specific PDE inhibitors erythro-9-[2-hydroxy-3-nonyl]adenine (EHNA; PDE2 inhibitor; 10 µM) [25], milrinone (Mil; PDE3 inhibitor; 10 µM) [10], [20] and rolipram (Rol; PDE4 inhibitor; 10 µM) [10]. The selectivity of these inhibitors for specific PDE families is well established, but also concentration dependent. Accordingly, we have used each of these compounds at concentrations that have been previously shown to be selective to their respective PDE family [3], [10], [11], [26], [27].

The effects of PDE2 inhibition with EHNA on AP firing in mouse SAN and right atrial myocytes are presented in Figure 3 (see also Tables S3 and S4). EHNA increased (*P*<0.05) spontaneous AP frequency in SAN myocytes from 137±7 to 160±6 APs/min (Figure 3B). In addition, EHNA increased (*P*<0.05) DD slope (28.9±1.4 mV/s in control vs. 41.2 mV/s in EHNA; Figure 3C) and APD_50_ (33.6±3.8 ms in control vs. 39.6±4.1 ms in EHNA; Figure 3D). EHNA also increased (*P*<0.05) AP duration in mouse right atrial myocytes (Figure 3F). Specifically, APD_50_ was increased from 9.4±1.1 to 12±1.1 ms, APD_70_ was increased from 17.5±2.3 to 22.3±2.3 ms and APD_90_ was increased from 45±4.6 to 50.5±5.2 ms. The effects of EHNA were reversed upon washout. These data demonstrate that PDE2 constitutively regulates AP firing in mouse SAN and atrial myocytes in basal conditions.

The effects of the PDE3 inhibitor Mil on AP firing in mouse SAN and right atrial myocytes are illustrated in Figure 4 (see also Tables S5 and S6). Mil increased (*P*<0.05) spontaneous SAN myocyte AP frequency from 135±6 to 169±6 APs/min (Figure 4B). Mil also increased (*P*<0.05) DD slope from 28.5±3.4 to 49.9±4.5 mV/s (Figure 4C) and APD_50_ from 37±5.4 to 46.2±5.5 ms (Figure 4D) in SAN myocytes. These effects of Mil on SAN myocytes were fully reversible. In contrast to the SAN, Mil had no effect on AP properties in mouse right atrial myocytes. APD_50_ (8.9±1 vs. 9.4±1), APD_70_ (16.9±2 vs. 18.2±2.3) and APD_90_ (45.7±4.2 vs. 46±4.3) were not different (*P* = 0.79; Figure 4F) upon application of Mil. These data indicate that the mouse SAN has constitutive PDE3 activity that regulates spontaneous AP firing but that PDE3 inhibition does not modulate atrial AP properties in basal conditions.

The effects of the PDE4 inhibitor Rol on AP firing in mouse SAN and right atrial myocytes were measured next (Figure 5, Tables S7 and S8). Rol increased (*P*<0.05) spontaneous AP frequency in SAN myocytes from 130±8 to 170±9 APs/min (Figure 5B). In addition, Rol increased (*P*<0.05) DD slope (23.9±1.8 mV/s vs. 44.3±4.3 mV/s; Figure 5C) and APD_50_ (37.5±5 ms vs. 47.8±6.3 ms; Figure 5D) in SAN myocytes. Rol also increased (*P*<0.05) AP duration in right atrial myocytes (Figure 5F). Specifically, APD_50_ was increased from 9.4±1.7 to 12.9±2 ms, APD_70_ was increased from 19.9±3.4 to 27.4±3.9 ms, and APD_90_ was increased from 51.1±5.9 to 59.1±5.5 ms. These data show that constitutive PDE4 activity contributes to the regulation of AP firing in mouse SAN and atrial myocytes.

### Effects of PDE inhibition on L-type Ca^2+^ current in SAN and right atrial myocytes

Numerous studies have demonstrated that I_Ca,L_ is a major target of regulation by PDEs, including PDE2, 3 and 4 [10], [11], [25], [27], [28] Furthermore, I_Ca,L_ is a critical determinant of spontaneous AP firing in the SAN [15]–[17] as well as AP duration in working atrial and ventricular myocytes [18], [29], [30]. Thus, we next studied the effects of PDE inhibitors on I_Ca,L_ in mouse SAN and right atrial myocytes. For comparison purposes, we also measured I_Ca,L_ in right ventricular myocytes. Importantly, some differences exist in SAN, atrial and ventricular I_Ca,L_. Specifically, I_Ca,L_ in SAN and atrial myocytes is generated by both Ca_V_1.2 and Ca_V_1.3 channel isoforms [16], [17]. This causes total I_Ca,L_ to activate between −60 and −50 mV and I_Ca,L_ I–V curves to peak between −10 and 0 mV. In contrast, ventricular I_Ca,L_ is determined only by Ca_V_1.2 channels, activates positive to −40 mV and peaks at approximately +10 mV.

The effects of global PDE inhibition with IBMX (100 µM) on SAN and right atrial myocyte I_Ca,L_ are illustrated in Figure 6. Summary I–V curves demonstrate that IBMX increased (*P*<0.05) peak I_Ca,L_ density from −4.4±0.3 to −11.2±1 (Figure 6B) in SAN myocytes. The effects of IBMX on SAN myocyte I_Ca,L_ were further studied using steady state conductance analysis (Figure 6C). IBMX increased (*P*<0.05) I_Ca,L_ maximum conductance (G_max_) from 103.9±6.4 to 225.1±5.9 pS/pF and shifted (*P*<0.05) the V_1/2(act)_ from −28.4±0.4 to −33.3±0.5 mV. There was no change (*P* = 0.588) in slope factor (6.4±0.6 mV in control vs. 6.3±0.3 mV in IBMX). Similarly, in right atrial myocytes, IBMX increased peak I_Ca,L_ density from −3.9±0.2 to −9.7±0.4 pA/pF (Figure 6E). Steady state conductance analysis in atrial myocytes (Figure 6F) shows that IBMX increased (*P*<0.05) I_Ca,L_ G_max_ from 112.8±8.8 to 195.8±10.4 pS/pF and shifted (*P*<0.05) the V_1/2(act)_ from −9.1±1.2 to −18.7±1.1 mV.

The effects of IBMX (100 µM) on I_Ca,L_ in right ventricular myocytes are illustrated in Figure S1. Summary I–V curves show that IBMX increased (*P*<0.05) peak I_Ca,L_ density from −4.9±0.7 to −11.2±0.9 pA/pF (Figure S1B) in ventricular myocytes. Furthermore, I_Ca,L_ G_max_ in ventricular myocytes was increased (*P*<0.05) from 115±15.2 to 197.1±23 pS/pF and V_1/2(act)_ was shifted (*P*<0.05) from −8.9±0.8 to −15.4±1 mV (Figure S1C) in IBMX. Thus, global PDE inhibition potently, and similarly, increases basal I_Ca,L_ in all regions of the myocardium we have investigated.

Next we measured the effects of the PDE2 inhibitor EHNA (10 µM) on SAN and right atrial myocyte I_Ca,L_ (Figure 7). In SAN myocytes EHNA increased (*P*<0.05) peak I_Ca,L_ density from −4.7±0.4 to −6.1±0.5 pA/pF (Figure 7B). Steady state conductance analysis illustrates that EHNA increased (*P*<0.05) I_Ca,L_ G_max_ from 106.0±7.7 to 125.5±5.2 pS/pF and shifted the V_1/2(act)_ from −25.1±1.5 to −28.5±1.6 mV (Figure 7C). In right atrial myocytes EHNA increased (*P*<0.05) I_Ca,L_ density from −3.4±0.2 to −4.9±0.5 pA/pF (Figure 7E). Furthermore, I_Ca,L_ G_max_ in right atrial myocytes was increased (*P*<0.05) from 72.8±2.3 to 99.9±3.2 pS/pF by EHNA and the V_1/2(act)_ was shifted (*P*<0.05) from −16.0±1.2 to −18.7±1.4 mV (Figure 7F). These data are consistent with the effects of EHNA on AP properties in SAN and atrial myocytes.

In contrast to SAN and atrial myocytes, EHNA had no effect on right ventricular myocyte I_Ca,L_ (Figure S2). Summary I–V curves (Figure S2B) show no change (*P* = 0.92) in I_Ca,L_ density upon application of EHNA. Steady state conductance analysis (Figure S2C) shows that I_Ca,L_ G_max_ (76.3±2.6 pS/pF in control vs. 76.1±2.9 pS/pF in EHNA) and V_1/2(act)_ (−13±1 mV in control vs. −14.4±1.2) were not altered (*P* = 0.85) in the presence of EHNA. Thus, although selective PDE2 inhibition does not affect ventricular myocyte I_Ca,L_, it does increase I_Ca,L_ in SAN and atrial myocytes.

The effects of the PDE3 inhibitor Mil (10 µM) on SAN and right atrial I_Ca,L_ are illustrated in Figure 8. Consistent with its effects on spontaneous AP firing, Mil increased (*P*<0.05) peak I_Ca,L_ density in SAN myocytes from −3.8±0.5 to −6.3±0.2 pA/pF (Figure 8B). Also, I_Ca,L_ G_max_ was increased (*P*<0.05) from 110.7±5.8 to 160.4±3.3 pS/pF and V_1/2(act)_ was shifted (*P*<0.05) from −16.8±2.3 to −18.4±2.1 mV by Mil (Figure 8C). In contrast to SAN myocytes, and in agreement with the absence of effects of PDE3 inhibition on right atrial AP duration, Mil had no effect on right atrial I_Ca,L_. Summary I–V curves (Figure 8E) show no change (*P* = 0.66) in peak I_Ca,L_ density upon application of Mil. Furthermore, I_Ca,L_ G_max_ (95.1±2.9 pS/pF in control vs. 100.9±2.3) and V_1/2(act)_ (−10.4±1.2 mV in control vs. −9.9±0.9 mV in Mil) were not altered following PDE3 inhibition in atrial myocytes.

The effects of Mil on right ventricular myocyte I_Ca,L_ are illustrated in Figure S3. Summary I–V curves show that Mil had no effect (*P* = 0.63) on peak I_Ca,L_ density (Figure S3B). Also, I_Ca,L_ G_max_ (87.2±1.8 pS/pF in control vs. 91.3±1.4 pS/pF in Mil) and V_1/2(act)_ (−8.8±0.6 mV in control vs. −9.2±0.4 mV in Mil) were not affected (*P* = 0.52) by Mil (Figure S3C). Together, these data show that PDE3 inhibition alone markedly increases I_Ca,L_ in the specialized pacemaker myocytes of the SAN, but has no effect on I_Ca,L_ in working atrial or ventricular myocytes.

The effects of PDE4 inhibition with Rol (10 µM) on SAN and right atrial myocyte I_Ca,L_ are presented in Figure 9. In SAN myocytes Rol increased (*P*<0.05) peak I_Ca,L_ density from −4.2±0.1 to −8.1±1.1 pA/pF (Figure 9B). Rol also increased (*P*<0.05) I_Ca,L_ G_max_ from 99.2±7.2 to 188.3±2.8 pS/pF and shifted (*P*<0.05) the V_1/2(act)_ from −18.1±3.1 to −20.2±0.7 mV (Figure 9C). In right atrial myocytes Rol increased (*P*<0.05) peak I_Ca,L_ density from −4.5±0.4 to −7.5±0.7 pA/pF (Figure 9E). Furthermore, Rol increased (*P*<0.05) I_Ca,L_ G_max_ from 130.8±0.7 to 176.9±1.0 pS/pF and shifted (*P*<0.05) the V_1/2(act)_ from −7.1±0.2 to −12.3±0.2 mV (Figure 9F). These effects of PDE4 inhibition on I_Ca,L_ are consistent with the ability of Rol to augment spontaneous AP firing in SAN myocytes and AP duration in atrial myocytes.

In contrast to SAN and atrial myocytes, Rol had no effect on right ventricular myocyte I_Ca,L_ (Figure S4). Summary I–V curves illustrate no change (*P* = 0.13) in peak I_Ca,L_ density following application of Rol. In addition, neither I_Ca,L_ G_max_ (98.5±1.5 pS/pF in control vs. 95.1±1.9 pS/pF in Rol) or V_1/2(act)_ (−9.1±0.4 mV in control vs. −8.9±0.6) were modulated (*P* = 0.53) by Rol. Thus, selective PDE4 inhibition elicits increases in I_Ca,L_ in SAN and atrial myocytes, but not in ventricular myocytes.

The observation that selective PDE inhibitors, when given alone, have no effect on basal I_Ca,L_ in ventricular myocytes is consistent with previous studies in mice and rats, which have shown that selective inhibitors must be given in combination to augment basal I_Ca,L_ [10], [11]. We have confirmed this pattern in our experiments by demonstrating that combined inhibition of PDE3 and PDE4 with Mil and Rol increases right ventricular myocyte I_Ca,L_ (Figure S5). Summary I–V curves illustrate that Mil + Rol increased peak I_Ca,L_ density (−4.5±0.3 pA/pF in control vs. −6.7±0.6 pA/pF in Mil + Rol; *P*<0.05), increased I_Ca,L_ G_max_ (84.8±1.5 pS/pF in control vs. 105.5±3.9 pS/pF in Mil + Rol; *P*<0.05) and shifted the V_1/2(act)_ (−11.2±0.5 mV in control vs. −20.8±1.1 mV in Mil + Rol; *P*<0.05) compared to control. Thus, our data illustrating the effects of global and specific PDE inhibitors on ventricular I_Ca,L_ fully agree with previous studies, which supports the conclusion that specific PDE inhibitors have distinct effects in different regions of the myocardium. These ventricular I_Ca,L_ data also confirm that the PDE inhibitors we have used are selective at the doses we have studied. We also measured the effects of combined PDE3/4 inhibition on AP firing and I_Ca,L_ in SAN and right atrial myocytes. We observed that Mil + Rol augments AP firing and I_Ca,L_ in SAN and atrial myocytes similarly to IBMX (data not shown).

## Discussion

PDEs are critical regulators of cAMP and cGMP levels in cardiomyocytes, representing the primary enzymes responsible for cyclic nucleotide degradation, and acting as the only counter balance to the mechanisms responsible for cyclic nucleotide production (adenylyl and guanylyl cyclases) [1], [2]. In this study, we have measured the effects of global (IBMX) and specific (EHNA, Mil, Rol) PDE inhibitors on AP firing and I_Ca,L_ in mouse SAN and atrial myocytes. We also measured the effects of these inhibitors on mouse ventricular I_Ca,L_ in order to make direct comparisons between the three myocyte types. This is the first direct comparison of these effects in the mouse myocardium. The main finding of these experiments is that PDE effects on AP firing and basal I_Ca,L_ are distinct in the different regions of the myocardium.

Our AP data clearly demonstrate that global PDE inhibition with IBMX potently increases spontaneous AP frequency in mouse SAN myocytes in association with increases in DD slope and APD_50_. Similarly IBMX markedly increased AP duration at 50, 70 and 90% repolarization in mouse atrial myocytes. These data show that there is a high level of constitutive PDE activity that regulates AP properties in the SAN and atrial myocardium; however, subsequent experiments with specific PDE inhibitors revealed important differences between the two cell types. Specifically, in mouse SAN myocytes, EHNA, Mil and Rol each increased spontaneous AP frequency on their own, indicating that PDE2, PDE3 and PDE4 all contribute to the regulation of AP properties in basal conditions. In contrast, in mouse atrial myocytes, only EHNA and Rol were able to increase AP duration whereas Mil had no effect, indicating that constitutive PDE2 and PDE4, but not PDE3 activity, regulates atrial AP properties.

Similar trends were also observed for I_Ca,L_ in SAN and atrial myocytes. Specifically, IBMX increased basal I_Ca,L_ by approximately 185% in SAN myocytes and 140% in atrial myocytes. Consistent with our AP measurements, SAN myocyte I_Ca,L_ was increased by each of the selective inhibitors EHNA (∼31%), Mil (∼66%) and Rol (∼93%). In atrial myocytes only EHNA (∼38%) and Rol (∼72%) increased basal I_Ca,L_. Mil had no significant effect on right atrial I_Ca,L_ once again confirming a key difference between SAN and atrial myocytes in terms of which specific PDE inhibitors are able to modulate basal AP properties and I_Ca,L_ in mice.

For further comparison, we measured the effects of IBMX on basal I_Ca,L_ in mouse right ventricular myocytes and observed increases of approximately 130%. In agreement with prior studies [8], [10], [11], but in contrast to our findings in SAN and atrial myocytes, none of the selective PDE inhibitors we tested had any effect on basal I_Ca,L_ in ventricular myocytes. Only when Mil and Rol were applied together was ventricular I_Ca,L_ increased as also noted previously [11]. Thus, although IBMX potently and similarly increases I_Ca,L_ in all regions of the myocardium, the ability of selective PDE inhibitors (EHNA, Mil or Rol) to modulate basal I_Ca,L_ is strikingly different in SAN, atrial and ventricular myocytes.

We also measured the mRNA expression of specific isoforms from the PDE2, PDE3 and PDE4 family in SAN, right atrial and right ventricular myocardium using quantitative PCR. These data show that PDE2A, PDE3A, PDE3B, PDE4A, PDE4B and PDE4D are all expressed in each region of the myocardium and that several differences exist in expression levels between SAN, right atrium and right ventricular free wall. Note that these specific isoforms were chosen based on prior studies that have shown these same isoforms are detectable in atrial and ventricular myocardium or isolated ventricular myocytes [7], [11], [31]. Comparing our electrophysiological data with our mRNA expression data suggests the differences in ability of specific PDE inhibitors to modulate AP firing and basal I_Ca,L_ in the different regions of the myocardium are not strictly related to differences in expression. For example, PDE2A is similarly expressed in each region of the myocardium yet PDE2 inhibition with EHNA augments I_Ca,L_ in SAN and atrial myocytes, but not ventricular myocytes. Also, PDE3A (thought to be the main PDE3 isoform in cardiac myocytes [32], [33]) is lowest in the SAN and highest in the ventricular myocardium; however, PDE3 inhibition with Mil only modulates basal I_Ca,L_ in SAN myocytes. Thus, it is likely that factors other than expression levels contribute importantly to the different effects of selective PDE inhibitors in different regions of the heart. These could include differences in subcellular localization of PDEs by anchoring proteins, differences in basal adenylyl and guanylyl cyclase activities, and differences in activity of PDE regulatory proteins in SAN, atrial and ventricular myocytes. It should also be noted that mRNA levels do not necessarily correlate with protein levels and/or enzymatic activity. Our mRNA measurements were done at the tissue level thus, in addition to myocytes, other cell types, such as cardiac fibroblasts, would be expected to be present in these samples and may contribute to total PDE mRNA levels. Further studies will therefore be required to better understand the basis for the differences in effects of PDE inhibitors in each region of the myocardium.

As our study represents the first direct comparison of different PDE inhibitors on electrophysiological responses in SAN, atrial and ventricular myocytes in mice it is useful to compare our data with those reported in studies that have mainly used other species. Few studies have characterized the effects of PDE inhibitors in the specialized SAN; however, one recent report [34] did demonstrate the effects of various PDE inhibitors on spontaneous AP frequency in rabbit SAN myocytes. This study showed that PDE3 was the major constitutively active PDE in the basal state in rabbit, with Mil increasing AP frequency almost as effectively as IBMX. Other PDE inhibitors, including EHNA and Rol had much smaller effects. This is different from what we have observed in the mouse in which Mil and Rol each had similar stimulatory effects on AP frequency and where the stimulatory effect of EHNA, although smaller than Mil and Rol, was still robust. Other studies have also shown that PDE3 inhibitors increase SAN AP frequency in rabbits [35] and guinea pigs [36]; however, these studies did not compare these effects with inhibitors of other PDE families. Our data showing that PDE3 and PDE4 inhibitors can similarly increase AP frequency and I_Ca,L_ in mouse SAN myocytes are consistent with studies showing these inhibitors also increase beating rate in mouse and rat atrial preparations [37].

Several studies have measured the effects of selective PDE inhibitors on I_Ca,L_ in human atrial myocytes and these findings can be compared to our observations in mouse right atrial myocytes. Similar to our findings in mouse atrial myocytes, inhibition of PDE2 with EHNA potentiates basal I_Ca,L_ in human atrial myocytes [27] indicating that PDE2 regulates basal I_Ca,L_ in both mouse and human atrium. In contrast, whereas we found no effect of Mil on basal I_Ca,L_ in mouse atrial myocytes, PDE3 inhibition does increase basal I_Ca,L_ in human atrial myocytes [28], [38]. Recently, PDE4 has also been shown to regulate basal cAMP levels in human atrial myocytes using a fluorescence resonance energy transfer based reporter system [39] suggesting that PDE4 likely regulates basal I_Ca,L_ in human atrium as it does in mice. This would be consistent with data indicating that the role of PDE4 in cAMP regulation is conserved between human and rodent hearts [31].

Our experiments have focused on the PDE2, 3 and 4 families and on the role of I_Ca,L_ in mediating changes in AP properties in SAN and atrial myocytes. This was done in order to facilitate comparisons and highlight differences with the effects of PDE inhibitors on ventricular I_Ca,L_. Although the changes in AP properties we observed in SAN and atrial myocytes correlated very well with changes in I_Ca,L_ it is likely that other ionic mechanisms contribute as well. For example, the hyperpolarization-activated current (I_f_) contributes prominently to the DD in SAN myocytes and is well known to be directly modulated by cAMP [13], [15]. We have shown that PDE3 inhibition with Mil increases basal I_f_ in mouse SAN myocytes [20] and it has also been shown that IBMX increases I_f_ in rabbit SAN myocytes [40]. Also, the slope of the DD in SAN myocytes is partly determined by Ca^2+^ release from the SR and a subsequent activation of the Na^+^-Ca^2+^ exchanger (I_NCX_) [12]. This SR Ca^2+^ release mechanism is also cAMP sensitive and has been shown to be constitutively regulated by PDE3 [34]. Other possible targets include cyclic nucleotide sensitive delayed rectifier K^+^ currents, such as I_Ks_, which can be modulated by IBMX and Mil in guinea pig SAN myocytes [41]. It is not known if mouse SAN expresses I_Ks_, but it has been shown to express I_Kr_ channels that are thought to be cAMP sensitive [42]. Further studies will be needed to assess the possible contributions of each of these ionic currents to the changes in AP properties elicited by PDE inhibitors in mouse SAN. Interestingly, spontaneous AP firing in the SAN is also partially determined by a T-type Ca^2+^ current (I_Ca,T_) [15], [43], [44]. Numerous studies have shown that I_Ca,T_, including in SAN myocytes, is not modulated by cAMP [43], [45]–[48]; thus, it is unlikely that I_Ca,T_ is affected by PDE inhibitors.

It is also possible that other PDE families contribute to AP and ionic current regulation in SAN and atrial myocytes. For example, PDE1 and PDE5 have been shown to be expressed in the heart and to play functional roles in some settings [1], [7], [49], [50]. More recently PDE8 has also been identified in ventricular myocardium [7]. Whether these PDE families have distinct roles in SAN or atrial myocytes in mice is yet to be determined.

In summary, we have characterized the effects of inhibitors of PDE2, PDE3 and PDE4 on AP properties in mouse SAN and atrial myocytes and directly compared the effects of these inhibitors on basal I_Ca,L_ in SAN, right atrial and right ventricular myocytes. Although IBMX potently increased I_Ca,L_ in all cell types our data demonstrate that each region of the myocardium displays a unique pattern of response to family specific PDE inhibitors. SAN myocytes were responsive to each specific inhibitor tested, while atrial myocytes were responsive to PDE2 and PDE4, but not PDE3 inhibitors. Ventricular myocytes, on the other hand, do not respond to any family specific PDE inhibitors when given alone. These differences may be important when considering cyclic nucleotide regulation of ion channels in different regions of the heart. It is now known that PDE signaling in the heart is complex, based on the hypothesis that PDEs can be compartmentalized to distinct subcellular regions. Our data show that another aspect of the complexity of PDE regulation in the heart is related to regional differences in the roles of specific PDE families on ion channel regulation in the SAN compared to the working (atrial and ventricular) myocardium.

## Supporting Information

Figure S1 **Effects of IBMX on L-type Ca^2+^ current in right ventricular myocytes.** **A.** Representative I_Ca,L_ recordings (at 0 mV from −40 mV) in right ventricular myocytes in control conditions, in the presence of IBMX (100 µM), and after IBMX washout. **B.** Summary I–V relationships for the effects of IBMX on right ventricular I_Ca,L_. **C.** Summary I_Ca,L_ conductance density plots for the effects of IBMX on right ventricular myocytes. Summary data are means ± SEM; *n* = 6 ventricular myocytes; *P<0.05 vs. control by paired Student's *t*-test.
(TIF)

Figure S2 **Effects of PDE2 inhibition with EHNA on L-type Ca^2+^ current in right ventricular myocytes.** **A.** Representative I_Ca,L_ recordings (at 0 mV from −40 mV) in right ventricular myocytes in control conditions, in the presence of EHNA (10 µM), and after EHNA washout. **B.** Summary I–V relationships for the effects of EHNA on right ventricular I_Ca,L_. **C.** Summary I_Ca,L_ conductance density plots for the effects of EHNA on right ventricular myocytes. Summary data are means ± SEM; *n* = 10 ventricular myocytes; EHNA had no effect on right ventricular I_Ca,L_ (paired Student's *t*-test).
(TIF)

Figure S3 **Effects of PDE3 inhibition with milrinone on L-type Ca^2+^ current in right ventricular myocytes.** **A.** Representative I_Ca,L_ recordings (at 0 mV from −40 mV) in right ventricular myocytes in control conditions, in the presence of Mil (10 µM), and after Mil washout. **B.** Summary I–V relationships for the effects of Mil on right ventricular I_Ca,L_. **C.** Summary I_Ca,L_ conductance density plots for the effects of Mil on right ventricular myocytes. Summary data are means ± SEM; *n* = 5 ventricular myocytes; Mil had no effect on right ventricular I_Ca,L_ (paired Student's *t*-test).
(TIF)

Figure S4 **Effects of PDE4 inhibition with rolipram on L-type Ca^2+^ current in right ventricular myocytes.** **A.** Representative I_Ca,L_ recordings (at 0 mV from −40 mV) in right ventricular myocytes in control conditions, in the presence of Rol (10 µM), and after Rol washout. **B.** Summary I–V relationships for the effects of Rol on right ventricular I_Ca,L_. **C.** Summary I_Ca,L_ conductance density plots for the effects of Rol on right ventricular myocytes. Summary data are means ± SEM; *n* = 5 ventricular myocytes; Rol had no effect on right ventricular I_Ca,L_ (paired Student's *t*-test).
(TIF)

Figure S5 **Effects of PDE3 and PDE4 inhibition with milrinone and rolipram on L-type Ca^2+^ current in right ventricular myocytes.** **A.** Representative I_Ca,L_ recordings (at 0 mV from −40 mV) in right ventricular myocytes in control conditions, in the presence of Mil + Rol (10 µM each), and after drug washout. **B.** Summary I–V relationships for the effects of Mil + Rol on right ventricular I_Ca,L_. **C.** Summary I_Ca,L_ conductance density plots for the effects of Mil + Rol on right ventricular myocytes. Summary data are means ± SEM; *n* = 8 ventricular myocytes; *P<0.05 vs. control by paired Student's *t*-test.
(TIF)

Appendix S1 **Supplemental materials and methods.**
(PDF)

Table S1 **Effects of IBMX on spontaneous action potential parameters in isolated mouse SAN myocytes.**
(PDF)

Table S2 **Effects of IBMX on stimulated action potential parameters in isolated mouse right atrial myocytes.**
(PDF)

Table S3 **Effects of EHNA on spontaneous action potential parameters in isolated mouse SAN myocytes.**
(PDF)

Table S4 **Effects of EHNA on stimulated action potential parameters in isolated mouse right atrial myocytes.**
(PDF)

Table S5 **Effects of milrinone on spontaneous action potential parameters in isolated mouse SAN myocytes.**
(PDF)

Table S6 **Effects of milrinone on stimulated action potential parameters in isolated mouse right atrial myocytes.**
(PDF)

Table S7 **Effects of rolipram on spontaneous action potential parameters in isolated mouse SAN myocytes.**
(PDF)

Table S8 **Effects of rolipram on stimulated action potential parameters in isolated mouse right atrial myocytes.**
(PDF)
