# Supplementary figures and images for: Distinct Patterns of Constitutive Phosphodiesterase Activity in Mouse Sinoatrial Node and Atrial Myocardium
Source: PLoS One. 2012 Oct 15;7(10):e47652. doi: 10.1371/journal.pone.0047652 (PMC3471891; doi:10.1371/journal.pone.0047652)

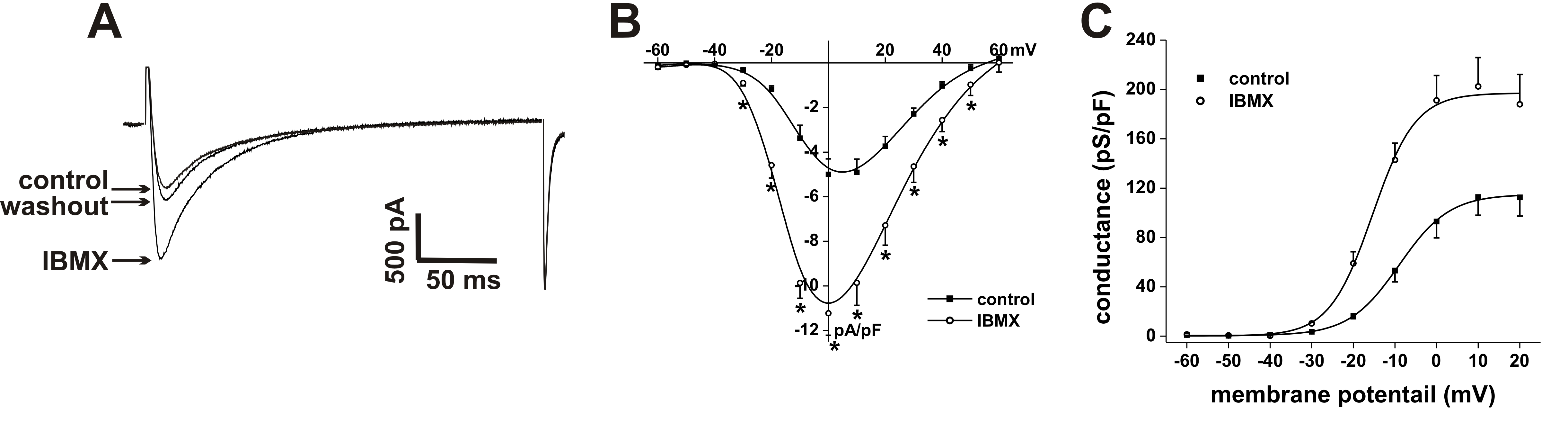

Supplement: Figure S1 — Effects of IBMX on L-type Ca2+ current in right ventricular myocytes. A. Representative ICa,L recordings (at 0 mV from −40 mV) in right ventricular myocytes in control conditions, in the presence of IBMX (100 µM), and after IBMX washout. B. Summary I–V relationships for the effects of IBMX on right ventricular ICa,L. C. Summary ICa,L conductance density plots for the effects of IBMX on right ventricular myocytes. Summary data are means ± SEM; n = 6 ventricular myocytes; *P<0.05 vs. control by paired Student's t-test. (TIF) [file pone.0047652.s001.tif]

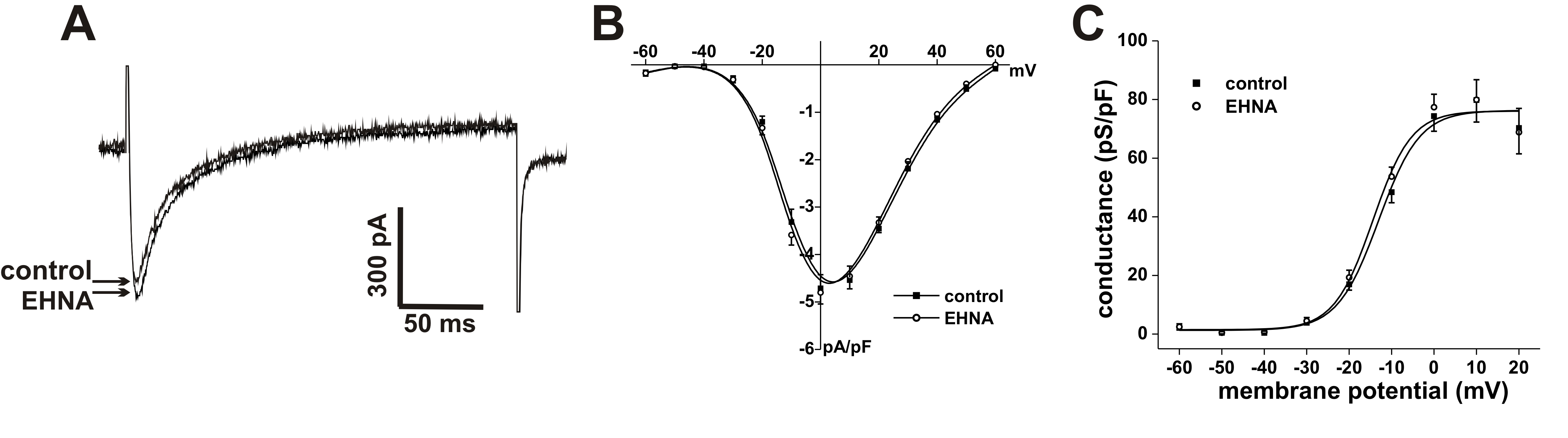

Supplement: Figure S2 — Effects of PDE2 inhibition with EHNA on L-type Ca2+ current in right ventricular myocytes. A. Representative ICa,L recordings (at 0 mV from −40 mV) in right ventricular myocytes in control conditions, in the presence of EHNA (10 µM), and after EHNA washout. B. Summary I–V relationships for the effects of EHNA on right ventricular ICa,L. C. Summary ICa,L conductance density plots for the effects of EHNA on right ventricular myocytes. Summary data are means ± SEM; n = 10 ventricular myocytes; EHNA had no effect on right ventricular ICa,L (paired Student's t-test). (TIF) [file pone.0047652.s002.tif]

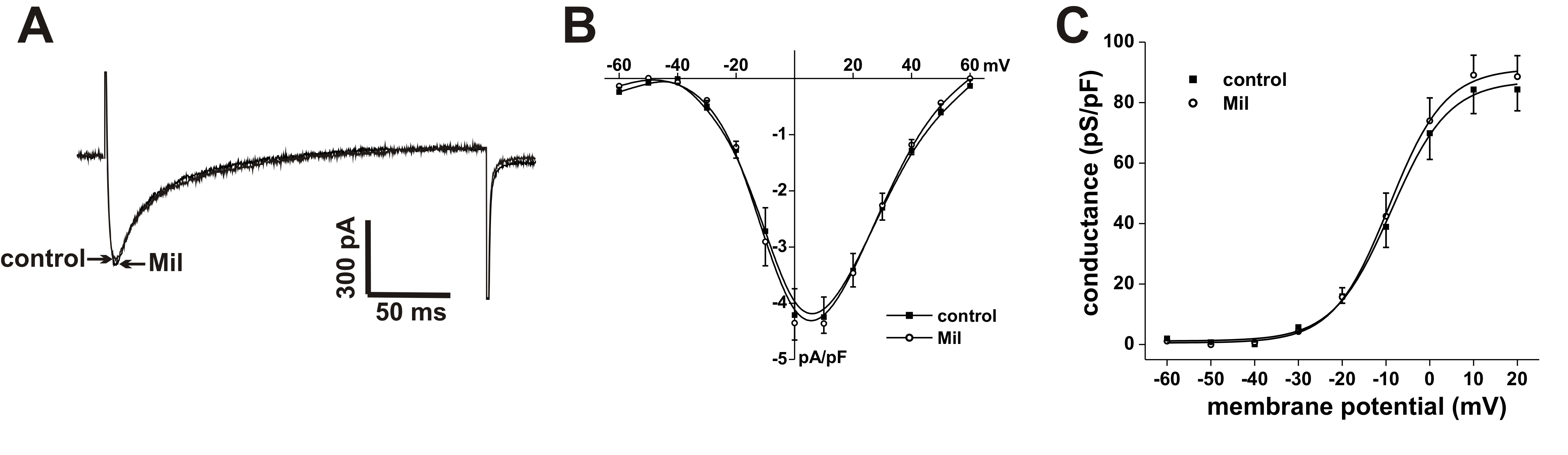

Supplement: Figure S3 — Effects of PDE3 inhibition with milrinone on L-type Ca2+ current in right ventricular myocytes. A. Representative ICa,L recordings (at 0 mV from −40 mV) in right ventricular myocytes in control conditions, in the presence of Mil (10 µM), and after Mil washout. B. Summary I–V relationships for the effects of Mil on right ventricular ICa,L. C. Summary ICa,L conductance density plots for the effects of Mil on right ventricular myocytes. Summary data are means ± SEM; n = 5 ventricular myocytes; Mil had no effect on right ventricular ICa,L (paired Student's t-test). (TIF) [file pone.0047652.s003.tif]

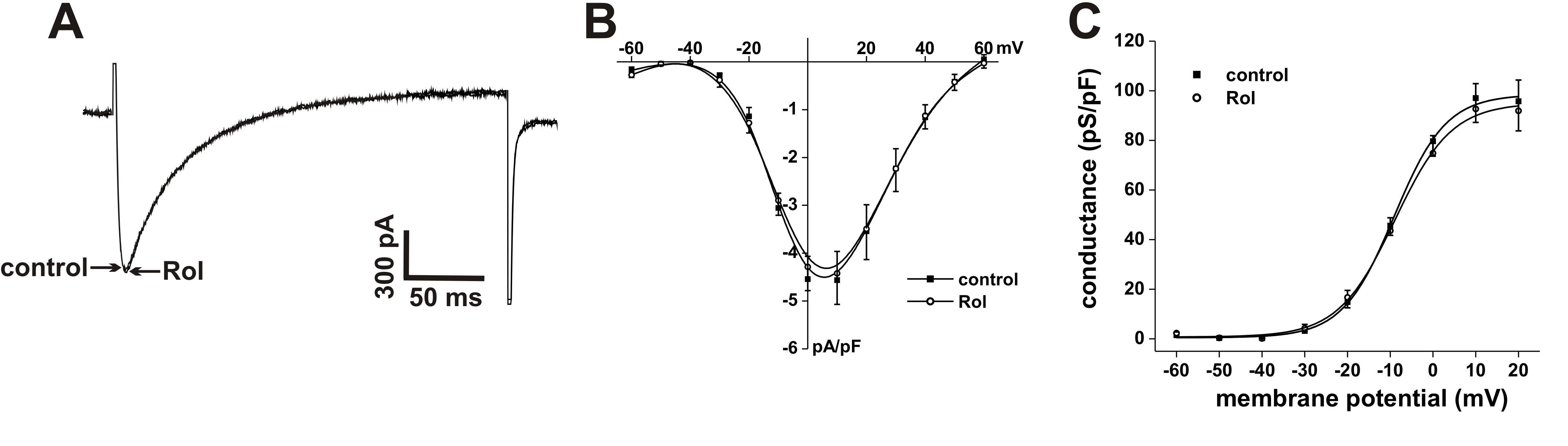

Supplement: Figure S4 — Effects of PDE4 inhibition with rolipram on L-type Ca2+ current in right ventricular myocytes. A. Representative ICa,L recordings (at 0 mV from −40 mV) in right ventricular myocytes in control conditions, in the presence of Rol (10 µM), and after Rol washout. B. Summary I–V relationships for the effects of Rol on right ventricular ICa,L. C. Summary ICa,L conductance density plots for the effects of Rol on right ventricular myocytes. Summary data are means ± SEM; n = 5 ventricular myocytes; Rol had no effect on right ventricular ICa,L (paired Student's t-test). (TIF) [file pone.0047652.s004.tif]

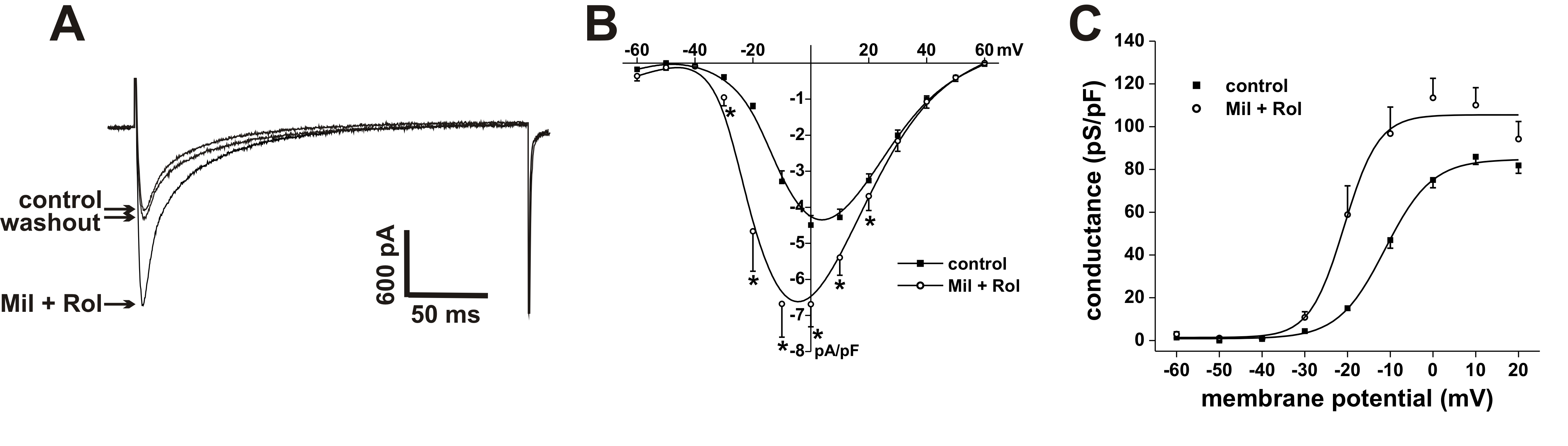

Supplement: Figure S5 — Effects of PDE3 and PDE4 inhibition with milrinone and rolipram on L-type Ca2+ current in right ventricular myocytes. A. Representative ICa,L recordings (at 0 mV from −40 mV) in right ventricular myocytes in control conditions, in the presence of Mil + Rol (10 µM each), and after drug washout. B. Summary I–V relationships for the effects of Mil + Rol on right ventricular ICa,L. C. Summary ICa,L conductance density plots for the effects of Mil + Rol on right ventricular myocytes. Summary data are means ± SEM; n = 8 ventricular myocytes; *P<0.05 vs. control by paired Student's t-test. (TIF) [file pone.0047652.s005.tif]
