# Supplementary material for: Distinct Patterns of Constitutive Phosphodiesterase Activity in Mouse Sinoatrial Node and Atrial Myocardium
Source: PLoS One. 2012 Oct 15;7(10):e47652. doi: 10.1371/journal.pone.0047652 (PMC3471891; doi:10.1371/journal.pone.0047652)
Supplement: Appendix S1 — Supplemental materials and methods. (PDF) [file pone.0047652.s006.pdf]

Distinct patterns of constitutive phosphodiesterase activity in mouse sinoatrial node and atrial myocardium

Supplemental Material

Supplemental Methods

Quantitative PCR

Quantitative gene expression studies were performed using an approach we [1] and others [2] have described in which SAN, right atrial and right ventricular free wall tissue samples were separately dissected for mRNA analysis. In order to have sufficient RNA 5 SAN samples were pooled for each individual trial [1,2]. Tissue samples were flash frozen in liquid nitrogen following dissection.

Intron spanning primers were designed for PDE2A, PDE3A, PDE3B, PDE4A, PDE4B and PDE4D as well as GAPDH (reference gene) and tested using Amplify 3 (software for simulating and testing polymerase chain reactions). Following synthesis (Sigma Genosys) primers were reconstituted to 100 nM in nuclease free water and stored at -20 C until experimental use. Primer sequences were as follows:

PDE2A: forward 5'-GGTGGCCTCGAAATCTGTGCTGG-3';
reverse 5'-GCATGCGCTGATAGTCCTTCCG-3'
Product size: 149 base pairs.

PDE3A: forward 5'-GGACAAACCAATTCTTGCTCCAGAACCC-3'
Reverse 5'-GATACCTGGCTCAGAATACGGCCAC-3'
Product size: 144 base pairs

PDE3B: forward 5'- CTTCACAAGGGATTGAGTGGCAGAACC-3'
Reverse 5'-CATCCATGACTTGAAACACTGACTTCTTGG-3'
Product size: 150 base pairs

PDE4A: forward 5’-TGGATGCCGTGTTCACAGACCTGG-3’

Reverse 5’-GTTCTCAAGCACAGACTCATCGTTGTAC-3’

Product size: 152 base pairs

PDE4C: forward 5’-CAGGAAAATGGTGATTGACATGGTGTTGG-3’

Reverse 5’-CGAAGAACCTGTATCCGGTCAGTATAG-3’

Produce size: 152 base pairs

PDE4D: forward 5’-GGTCATTGACATTGTCCTGGCGACAG-3’

Reverse 5’-CAGTGCACCATATTCTGAAGGACCTGG-3’

Product size: 159 base pairs

RNA was extracted in PureZOL^{TM} RNA isolation reagent according to kit instructions (Aurum^{TM} total RNA fatty and fibrous tissue kit, BioRad). Tissue was eluted in 30-40 µl of elution buffer (provided) from the spin column. RNA concentrations were determined using a Qubit fluorometer (Invitrogen) and first strand synthesis reactions were performed using the iScript^{TM} cDNA synthesis kit (BioRad) according to kit directives with 0.5 µg RNA template for right atrial and ventricular samples. SAN samples were not quantified as initial screening indicated that RNA template concentrations in these samples were limiting. Instead, total RNA extract obtained from SAN samples were used in first strand synthesis. A260/280 readings were also performed to evaluate the purity of RNA extractions prior to first strand synthesis. Lack of genomic DNA contamination was verified by reverse transcription (RT)-PCR using a no RT control.

SYBR green RT-qPCR was used to assess gene expression in 3-5 distinct pools of tissue. Following RNA extraction cDNA was synthesized for SAN tissue, right atrial tissue, and right

ventricular free wall tissue samples. 20 µl SYBR reactions were performed with 1 µl cDNA template. Varying concentrations were used to obtain data that crossed threshold for both sample types at similar cycles. Reactions were carried out using a CFX96™ Real-Time PCR Detection System (BioRad). Amplification conditions were as follows: 95^0C for 2 min to activate Taq polymerase, 35 cycles of denaturation at 95^0C for 30 sec, annealing using a gradient from 53-61^0C for 30 sec and extension at 72^0C or 1 min 30 sec. Melt curve analysis was performed from 65-95^0C every 0.5^0C increments.

Single amplicons with appropriate melting temperatures and sizes were detected. Data were expressed in the form 2^{-CT} x 100 *versus* GAPDH for all tissue samples. C_T values > 32 were eliminated due to lack of reproducibility [2]. Primers were used at a concentration of 10 nM.

Myocyte isolations

The procedures for isolating single pacemaker myocytes from the mouse sinoatrial node (SAN) have been described previously [3-6] and were as follows. Mice were administered a 0.2 ml intraperitoneal injection of heparin (1000 IU/ml) to prevent blood clotting. Following this, mice were anesthetized by isoflurane inhalation and then killed by cervical dislocation. The heart was excised into Tyrode's solution (35ºC) consisting of (in mmol/L) 140 NaCl, 5.4 KCl, 1.2 KH_2PO_4, 1.0 $MgCl_2$, 1.8 $CaCl_2$, 5.55 glucose, and 5 HEPES, with pH adjusted to 7.4 with NaOH. The sinoatrial node (SAN) region of the heart was isolated by separating the atria from the ventricles, cutting open the superior and inferior venae cavae, and pinning the tissue so that the crista terminalis could be identified. The SAN area is located in the intercaval region adjacent to the crista terminalis. This SAN region was cut into strips, which were transferred and rinsed in a

'low Ca^{2+}, Mg^{2+} free' solution containing (in mmol/L) 140 NaCl, 5.4 KCl, 1.2 KH_2PO_4, 0.2 $CaCl_2$, 50 taurine, 18.5 glucose, 5 HEPES and 1 mg/ml bovine serum albumin (BSA), with pH adjusted to 6.9 with NaOH. SAN tissue strips were digested in 5 ml of 'low Ca^{2+}, Mg^{2+} free' solution containing collagenase (type II, Worthington Biochemical Corporation), elastase (Worthington Biochemical Corporation) and protease (type XIV, Sigma Chemical Company) for 30 min. Then the tissue was transferred to 5 ml of modified KB solution containing (in mmol/L) 100 potassium glutamate, 10 potassium aspartate, 25 KCl, 10 KH_2PO_4, 2 $MgSO_4$, 20 taurine, 5 creatine, 0.5 EGTA, 20 glucose, 5 HEPES, and 0.1% BSA, with pH adjusted to 7.2 with KOH. The tissue was mechanically agitated using a wide-bore pipette. This procedure yielded individual SAN myocytes with cellular automaticity that was recovered after readapting the cells to a physiological concentration of Ca^{2+}. The identical enzymatic procedure was used on tissue dissected from the right atrial appendage to isolate working right atrial myocytes.

SAN myocytes were identified by their small spindle shape and ability to beat spontaneously in the recording chamber when superfused with normal Tyrode's solution. When patch-clamped, SAN myocytes always displayed spontaneous action potentials. The capacitance of single SAN myocytes was 20-35 pF, while that of right atrial myocytes was 45-60 pF.

Mouse right ventricular myocytes were isolated as using procedures described previously [7]. Briefly, Mice were administered a 0.2 ml intraperitoneal injection of heparin (1000 IU/ml) 10 min before heart excision. Mice were anesthetized by isoflurane inhalation, cervically dislocated and the heart was rapidly excised and placed in ice cold Tyrode's solution containing (in mmol/L): 140 NaCl, 5.4 KCl, 1 $CaCl_2$, 1 $MgCl_2$, 1 Na_2HPO_4, 10 HEPES, 10 glucose with pH adjusted to 7.4 with NaOH. Hearts were hung on a modified Langendorff apparatus and retrogradely perfused via the aorta with the following solutions: 5 min normal Tyrode's solution,

10 min Ca^{2+} free Tyrode's solution and 20 min with a Tyrode's solution containing 30 μM $CaCl_2$, 20 mM taurine, 0.1% BSA and 74 U/ml collagenase (Worthington Type II). All solutions were perfused at 37°C. At the end of the enzyme perfusion the right ventricular free wall was removed, placed in KB solution and gently agitated with a wide bore pipette to produce isolated, rod-shaped ventricular myocytes.

Solutions and electrophysiological protocols

Spontaneous action potentials (APs) and stimulated APs were recorded using the perforated patch-clamp technique [8] on single SAN and right atrial myocytes. L-type Ca^{2+} currents ($I_{Ca,L}$) were recorded by voltage clamping single SAN, right atrial or right ventricular myocytes using the patch-clamp technique in the whole cell configuration [9]. APs and $I_{Ca,L}$ were recorded at room temperature (22-23 ºC).

For recording APs the recording chamber was superfused with a normal Tyrode's solution (22 – 23ºC) containing (in mmol/L) 140 NaCl, 5 KCl, 1 $MgCl_2$, 1 $CaCl_2$, 10 HEPES, and 5 glucose, with pH adjusted to 7.4 with NaOH. The pipette filling solution contained (in mmol/L) 135 KCl, 0.1 $CaCl_2$, 1 $MgCl_2$, 5 NaCl, 10 EGTA, 4 Mg-ATP, and 10 HEPES, with pH adjusted to 7.2 with KOH. Amphotericin B (200 µg/ml) was added to this pipette solution to record APs with the perforated patch clamp technique.

For recording $I_{Ca,L}$ SAN and atrial myocytes were superfused with a modified Tyrode's solution (22 – 23ºC) containing the following (in mmol/L) 140 NaCl, 5.4 TEA-Cl, 2 $CaCl_2$, 1 $MgCl_2$, 10 HEPES, and 5 glucose with pH adjusted to 7.4 with NaOH. The pipette solution for $I_{Ca,L}$ contained (in mmol/L) 135 CsCl, 0.1 $CaCl_2$, 1 $MgCl_2$, 5 NaCl, 10 EGTA, 4 Mg-ATP, 6.6 Na-phosphocreatine, 0.3 Na-GTP and 10 HEPES, with pH adjusted to 7.2 with CsOH. In SAN

and atrial myocytes voltage gated Na^+ currents (I_{Na}) were blocked with lidocaine (0.3 mM) when recording $I_{Ca,L}$. This approach was used in order to record SAN and atrial $I_{Ca,L}$ from a holding potential of -60 mV due to the expression of $Ca_V1.2$ and $Ca_V1.3$ in these cells [10-12]. In right ventricular myocytes I_{Na} was inactivated with a voltage clamp step to -40 mV, which is possible because ventricular myocytes do not express $Ca_V1.3$ and $I_{Ca,L}$ activates positive to -40 mV. The external and pipette solutions used for measuring ventricular $I_{Ca,L}$ were the same as those used for SAN and atrial myocytes; however, the external Ca^{2+} concentration was 1 mmol/L. In all cases, $I_{Ca,L}$ was measured as the difference between peak current and the current at the end of the test pulse.

Micropipettes were pulled from borosilicate glass (with filament, 1.5 mm OD, 0.75 mm ID, Sutter Instrument Company) using a Flaming/Brown pipette puller (model p-87, Sutter Instrument Company). The resistance of these pipettes was 4 – 8 MΩ when filled with recording solution. Micropipettes were positioned with a micromanipulator (Burleigh PCS-5000 system) mounted on the stage of an inverted microscope (Olympus IX71). Seal resistance was 2 – 15 GΩ. For perforated patch clamp experiments access resistance was monitored for the development of capacitative transients upon sealing to the cell membrane with Amphotericin B in the pipette. Typically, access resistance became less than 25 MΩ within 5 min of sealing onto the cell, which was sufficient for recording spontaneous APs in current clamp mode. Data were digitized using a Digidata 1440 and pCLAMP 10 software (Molecular Devices) and stored on computer for *post hoc* analysis.

Spontaneous AP parameters, including the maximum diastolic potential (MDP), the slope of the diastolic depolarization (DD slope), the maximum AP upstroke velocity (Vmax), the AP overshoot and the AP duration at 50% repolarization (APD50) were analyzed as described

previously [4,13]. The DD slope was measured by fitting a straight line to the linear portion of this AP component [13].

$I_{Ca,L}$ activation kinetics were determined by calculating chord conductance (G) with the equation $G=I/(V_m-E_{rev})$, where V_m represents the depolarizing voltages and E_{rev} is the reversal potential estimated from the current-voltage relation of $I_{Ca,L}$. Maximum conductance (G_{max}) and $V_{1/2}$ of activation for $I_{Ca,L}$ were determined using the following function: $G=\{(V_m-V_{rev})\}\{G_{max}\}\{-1/[(1+\exp((V_m-V_{1/2})/k))+1]\}$.

Pharmacological compounds

All PDE inhibitors used in these experiments were obtained from Sigma Chemical Company.

References

1. Springer J, Azer J, Hua R, Robbins C, Adamczyk A, et al. (2012) The natriuretic peptides BNP and CNP increase heart rate and electrical conduction by stimulating ionic currents in the sinoatrial node and atrial myocardium following activation of guanylyl cyclase-linked natriuretic peptide receptors. J Mol Cell Cardiol 52:1122-1134.

2. Marionneau C, Couette B, Liu J, Li H, Mangoni ME, et al. (2005) Specific pattern of ionic channel gene expression associated with pacemaker activity in the mouse heart. J Physiol 562:223-234.

3. Lomax AE, Rose RA, Giles WR (2003) Electrophysiological evidence for a gradient of G protein-gated K+ current in adult mouse atria. Br J Pharmacol 140:576-584.

4. Mangoni ME, Nargeot J (2001) Properties of the hyperpolarization-activated current (I(f)) in isolated mouse sino-atrial cells. Cardiovasc Res 52:51-64.

5. Rose RA, Lomax AE, Kondo CS, Anand-Srivastava MB, Giles WR (2004) Effects of C-type natriuretic peptide on ionic currents in mouse sinoatrial node: a role for the NPR-C receptor. Am J Physiol Heart Circ Physiol 286:H1970-H1977.

6. Rose RA, Kabir MG, Backx PH (2007) Altered heart rate and sinoatrial node function in mice lacking the cAMP regulator phosphoinositide 3-kinase-gamma. Circ Res 101:1274-1282.

7. Rivard K, Grandy SA, Douillette A, Paradis P, Nemer M, et al. (2011) Overexpression of type 1 angiotensin II receptors impairs excitation-contraction coupling in the mouse heart. Am J Physiol Heart Circ Physiol 301:H2018-H2027.

8. Rae J, Cooper K, Gates P, Watsky M (1991) Low access resistance perforated patch recordings using amphotericin B. J Neurosci Methods 37:15-26.

9. Hamill OP, Marty A, Neher E, Sakmann B, Sigworth FJ (1981) Improved patch-clamp techniques for high-resolution current recording from cells and cell-free membrane patches. Pflugers Arch 391:85-100.

10. Mangoni ME, Couette B, Bourinet E, Platzer J, Reimer D, et al. (2003) Functional role of L-type Cav1.3 Ca2+ channels in cardiac pacemaker activity. Proc Natl Acad Sci U S A 100:5543-5548.

11. Zhang Z, Xu Y, Song H, Rodriguez J, Tuteja D, et al. (2002) Functional Roles of Ca(v)1.3 (alpha(1D)) calcium channel in sinoatrial nodes: insight gained using gene-targeted null mutant mice. Circ Res 90:981-987.

12. Zhang Z, He Y, Tuteja D, Xu D, Timofeyev V, et al. (2005) Functional roles of Cav1.3(alpha1D) calcium channels in atria: insights gained from gene-targeted null mutant mice. Circulation 112:1936-1944.

13. Honjo H, Boyett MR, Kodama I, Toyama J (1996) Correlation between electrical activity and the size of rabbit sino-atrial node cells. J Physiol 496 (Pt 3):795-808.
