# Supplementary material for: Distinct Patterns of Constitutive Phosphodiesterase Activity in Mouse Sinoatrial Node and Atrial Myocardium
Source: PLoS One. 2012 Oct 15;7(10):e47652. doi: 10.1371/journal.pone.0047652 (PMC3471891; doi:10.1371/journal.pone.0047652)
Supplement: Table S1 — Effects of IBMX on spontaneous action potential parameters in isolated mouse SAN myocytes. (PDF) [file pone.0047652.s007.pdf]

Table S1. Effects of IBMX on spontaneous action potential parameters in isolated mouse SAN myocytes.

	Control	IBMX	washout
Beating rate (APs/min)	138±10	193±14*	141±17
MDP (mV)	-65.3±1.1	-65.8±1.4	-66.8±1.2
DD slope (mV/s)	28.3±4	58.4±4.1*	29.6±2.4
V_{max} (V/s)	17.5±3.2	16.9±2.6	18.3±2.5
OS (mV)	7.1±3.9	10.3±3.8	7.9±3.8
APD_{50} (ms)	35.3±4.5	55.1±5.3*	39.5±6.5

IBMX was applied at 100 µM. MDP, maximum diastolic potential; DD slope, slope of the diastolic depolarization; V_{max}, maximum AP upstroke velocity; OS, overshoot; APD_{50}, action potential duration at 50% repolarization. Data are means ± SEM; *n*=11 SAN myocytes; *$P<0.05$ vs. control by one way ANOVA with a Tukey posthoc test.
