# Supplementary material for: Distinct Patterns of Constitutive Phosphodiesterase Activity in Mouse Sinoatrial Node and Atrial Myocardium
Source: PLoS One. 2012 Oct 15;7(10):e47652. doi: 10.1371/journal.pone.0047652 (PMC3471891; doi:10.1371/journal.pone.0047652)
Supplement: Table S2 — Effects of IBMX on stimulated action potential parameters in isolated mouse right atrial myocytes. (PDF) [file pone.0047652.s008.pdf]

Table S2. Effects of IBMX on stimulated action potential parameters in isolated mouse right atrial myocytes.

	Control	IBMX	washout
RMP (mV)	-76.9±0.2	-77.4±1	-77.4±1.5
V_{max} (V/s)	135.4±6	131.3±6.7	130.8±8.7
OS (mV)	53±3.9	52.5±5.7	47.5±4.6
APD_{50} (ms)	8.2±0.9	16.9±2.1*	9.3±1.0
APD_{70} (ms)	12.9±3.7	32.2±4.3*	17.4±2.1
APD_{90} (ms)	47.7±4.5	60.2±6.6*	44.7±4.8

IBMX was applied at 100 µM. RMP, resting membrane potential, V_{max}, maximum AP upstroke velocity; OS, overshoot; APD_{50}, action potential duration at 50% repolarization; APD_{70}, action potential duration at 70% repolarization; APD_{90}, action potential duration at 90% repolarization. Data are means ± SEM; *n*=13 SAN myocytes; *$P<0.05$ vs. control by one way ANOVA with a Tukey posthoc test.
