# Supplementary material for: Distinct Patterns of Constitutive Phosphodiesterase Activity in Mouse Sinoatrial Node and Atrial Myocardium
Source: PLoS One. 2012 Oct 15;7(10):e47652. doi: 10.1371/journal.pone.0047652 (PMC3471891; doi:10.1371/journal.pone.0047652)
Supplement: Table S3 — Effects of EHNA on spontaneous action potential parameters in isolated mouse SAN myocytes. (PDF) [file pone.0047652.s009.pdf]

Table S3. Effects of EHNA on spontaneous action potential parameters in isolated mouse SAN myocytes.

	Control	EHNA	washout
Beating rate (APs/min)	137±7	160±6*	136±7
MDP (mV)	-67.3±0.5	-67.6±0.7	-67.5±0.5
DD slope (mV/s)	28.9±1.4	41.2±1.7*	29.1±1.0
V_{max} (V/s)	15.1±5.2	15.5±4.1	13.8±4.2
OS (mV)	6.1±2.8	7.4±2.1	6.9±2.2
APD_{50} (ms)	33.6±3.8	39.6±4.1	34.4±3.1

EHNA (PDE2 inhibitor) was applied at 10 µM. MDP, maximum diastolic potential; DD slope, slope of the diastolic depolarization; V_{max}, maximum AP upstroke velocity; OS, overshoot; APD_{50}, action potential duration at 50% repolarization. Data are means ± SEM; *n*=6 SAN myocytes; **P*<0.05 vs. control by one way ANOVA with a Tukey posthoc test.
