# Supplementary material for: Distinct Patterns of Constitutive Phosphodiesterase Activity in Mouse Sinoatrial Node and Atrial Myocardium
Source: PLoS One. 2012 Oct 15;7(10):e47652. doi: 10.1371/journal.pone.0047652 (PMC3471891; doi:10.1371/journal.pone.0047652)
Supplement: Table S4 — Effects of EHNA on stimulated action potential parameters in isolated mouse right atrial myocytes. (PDF) [file pone.0047652.s010.pdf]

Table S4. Effects of EHNA on stimulated action potential parameters in isolated mouse right atrial myocytes.

	Control	EHNA	washout
RMP (mV)	-74.3±1.1	-74.6±1.1	-74.7±1.1
V_{max} (V/s)	123.1±7.3	120.6±8.1	119.1±8.1
OS (mV)	56.6±6.4	55.8±8.1	49.8±7.9
APD_{50} (ms)	9.4±1.1	12.0±1.1*	9.9±1.2
APD_{70} (ms)	17.5±2.3	22.3±2.3*	18.2±2.5
APD_{90} (ms)	45.0±4.6	50.5±5.2*	46.5±5.1

EHNA (PDE2 inhibitor) was applied at 10 µM. RMP, resting membrane potential, V_{max}, maximum AP upstroke velocity; OS, overshoot; APD_{50}, action potential duration at 50% repolarization; APD_{70}, action potential duration at 70% repolarization; APD_{90}, action potential duration at 90% repolarization. Data are means ± SEM; *n*=5 SAN myocytes; *$P<0.05$ vs. control by one way ANOVA with a Tukey posthoc test.
