# Supplementary material for: Distinct Patterns of Constitutive Phosphodiesterase Activity in Mouse Sinoatrial Node and Atrial Myocardium
Source: PLoS One. 2012 Oct 15;7(10):e47652. doi: 10.1371/journal.pone.0047652 (PMC3471891; doi:10.1371/journal.pone.0047652)
Supplement: Table S5 — Effects of milrinone on spontaneous action potential parameters in isolated mouse SAN myocytes. (PDF) [file pone.0047652.s011.pdf]

Table S5. Effects of milrinone on spontaneous action potential parameters in isolated mouse SAN myocytes.

	Control	Mil	washout
Beating rate (APs/min)	135±6	169±6*	136±8
MDP (mV)	-61.6±0.8	-61.0±1.0	-61.5±0.9
DD slope (mV/s)	28.5±3.4	49.9±4.5*	30.1±4.2
V_{max} (V/s)	11.3±1.6	14.2±2.3	10.8±3.4
OS (mV)	10.1±2.5	14.3±1.7	10.2±2.1
APD_{50} (ms)	37.0±5.4	46.2±5.5*	38.2±5.4

Milrinone (PDE3 inhibitor) was applied at 10 µM. MDP, maximum diastolic potential; DD slope, slope of the diastolic depolarization; V_{max}, maximum AP upstroke velocity; OS, overshoot; APD_{50}, action potential duration at 50% repolarization. Data are means ± SEM; n=9 SAN myocytes; *P<0.05 vs. control by one way ANOVA with a Tukey posthoc test.
