# Supplementary material for: Distinct Patterns of Constitutive Phosphodiesterase Activity in Mouse Sinoatrial Node and Atrial Myocardium
Source: PLoS One. 2012 Oct 15;7(10):e47652. doi: 10.1371/journal.pone.0047652 (PMC3471891; doi:10.1371/journal.pone.0047652)
Supplement: Table S6 — Effects of milrinone on stimulated action potential parameters in isolated mouse right atrial myocytes. (PDF) [file pone.0047652.s012.pdf]

Table S6. Effects of milrinone on stimulated action potential parameters in isolated mouse right atrial myocytes.

	Control	Mil	washout
RMP (mV)	-74.4±1.6	-75.1±1.6	-77.5±1.5
V_{max} (V/s)	126.6±8.7	130.2±8.5	125.8±9.2
OS (mV)	59.2±4.3	59.3±8.6	53.8±5.9
APD_{50} (ms)	8.9±1.0	9.4±1.0	9.8±1.3
APD_{70} (ms)	16.9±2.0	18.2±2.3	19.1±2.7
APD_{90} (ms)	45.7±4.2	46.0±4.3	46.1±4.9

Milrinone (PDE3 inhibitor) was applied at 10 µM. RMP, resting membrane potential, V_{max}, maximum AP upstroke velocity; OS, overshoot; APD_{50}, action potential duration at 50% repolarization; APD_{70}, action potential duration at 70% repolarization; APD_{90}, action potential duration at 90% repolarization. Data are means ± SEM; *n*=9 SAN myocytes; **P*<0.05 vs. control by one way ANOVA with a Tukey posthoc test.
