# Supplementary material for: Distinct Patterns of Constitutive Phosphodiesterase Activity in Mouse Sinoatrial Node and Atrial Myocardium
Source: PLoS One. 2012 Oct 15;7(10):e47652. doi: 10.1371/journal.pone.0047652 (PMC3471891; doi:10.1371/journal.pone.0047652)
Supplement: Table S7 — Effects of rolipram on spontaneous action potential parameters in isolated mouse SAN myocytes. (PDF) [file pone.0047652.s013.pdf]

Table S7. Effects of rolipram on spontaneous action potential parameters in isolated mouse SAN myocytes.

	Control	Rol	washout
Beating rate (APs/min)	130±8	170±9*	135±8
MDP (mV)	-66.4±1.0	-66.0±1.0	-67.6±0.8
DD slope (mV/s)	23.9±1.8	44.3±4.3*	29.8±2.3
V_{max} (V/s)	11.2±1.6	11.8±2.8	11.9±2.1
OS (mV)	9.5±0.8	9.8±1.48	8.9±1.3
APD_{50} (ms)	37.5±5.0	47.8±6.3*	39.9±5.1

Rolipram (PDE4 inhibitor) was applied at 10 µM. MDP, maximum diastolic potential; DD slope, slope of the diastolic depolarization; V_{max}, maximum AP upstroke velocity; OS, overshoot; APD_{50}, action potential duration at 50% repolarization. Data are means ± SEM; *n*=10 SAN myocytes; **P*<0.05 vs. control by one way ANOVA with a Tukey posthoc test.
