# Supplementary material for: Distinct Patterns of Constitutive Phosphodiesterase Activity in Mouse Sinoatrial Node and Atrial Myocardium
Source: PLoS One. 2012 Oct 15;7(10):e47652. doi: 10.1371/journal.pone.0047652 (PMC3471891; doi:10.1371/journal.pone.0047652)
Supplement: Table S8 — Effects of rolipram on stimulated action potential parameters in isolated mouse right atrial myocytes. (PDF) [file pone.0047652.s014.pdf]

Table S8. Effects of rolipram on stimulated action potential parameters in isolated mouse right atrial myocytes.

	Control	Rol	washout
RMP (mV)	-74.0±1.8	-74.6±1.4	-74.7±1.5
V_{max} (V/s)	149.1±8.8	152.5±5.5	153.2±5.3
OS (mV)	62.1±4.3	59.0±2.4	58.1±2.6
APD_{50} (ms)	9.4±1.7	12.9±2.0*	10.9±1.6
APD_{70} (ms)	19.9±3.4	27.4±3.9*	22.6±3.2
APD_{90} (ms)	51.1±5.9	59.1±5.5*	53.6±5.5

Rolipram (PDE4 inhibitor) was applied at 10 µM. RMP, resting membrane potential, V_{max}, maximum AP upstroke velocity; OS, overshoot; APD_{50}, action potential duration at 50% repolarization; APD_{70}, action potential duration at 70% repolarization; APD_{90}, action potential duration at 90% repolarization. Data are means ± SEM; *n*=7 SAN myocytes; **P*<0.05 vs. control by one way ANOVA with a Tukey posthoc test.
